# Datasets of essential oils from naturally formed and synthetically induced *Aquilaria malaccensis* agarwoods

**DOI:** 10.1016/j.dib.2019.104987

**Published:** 2019-12-12

**Authors:** Farah Adibah Abdul Kadir, Kamalrul Azlan Azizan, Roohaida Othman

**Affiliations:** a Faculty of Science and Technology, Universiti Kebangsaan Malaysia, 43600 UKM, Bangi, Selangor, Malaysia; b Institute of Systems Biology (INBIOSIS), Universiti Kebangsaan Malaysia, 43600 UKM, Bangi, Selangor, Malaysia

**Keywords:** *Aquilaria malaccensis*, Agarwood, Hydrodistillation, Gas chromatography mass spectrometer, Metabolomics

## Abstract

Agarwood is the highly valuable fragrant resin of the wounded *Aquilaria* spp. trees widely used in fragrances, medicines and incenses. Among the *Aquilaria* spp., *A. malaccensis* is the primary producer and is mainly found in Indonesia and Malaysia. In normal condition, agarwood is naturally formed in *Aquilaria* trees as a defense mechanism upon physical damage or microbial infection on the trees, which is a slow process that occurs over several years. The high demand in agarwood has spurred the development of various artificial inoculation methods where agarwood formation is synthetically induced in a shorter period of time. However, the synthetic induction method produces agarwood with aromas different from the naturally formed agarwood. To understand the changes in the agarwoods produced from different induction conditions, metabolite profiling of agarwood essential oil from *A. malaccensis* has been performed. The essential oils of healthy undamaged tree trunks and, naturally formed and synthetically induced agarwoods were obtained using hydrodistillation (HS) method and analysed using gas chromatography mass spectrometer (GC-MS). These data will provide valuable resources for chemical components of agarwood produced by the species in the genus *Aquilaria*.

**Table:** Specifications table

Subject area	Biology
More specific subject area	Metabolomics
Type of data	Raw data and Excel Table
How data was acquired	Metabolomics data were obtained using Perkin Elmer Clarus 600 GC-MS with autosampler
Data format	Normalised dataset is tabulated in MS excel (.xlsl format)
Experimental factors	Samples were obtained from healthy, undamaged *A. malaccensis* tree as the control. The trees containing the agarwood resins were those that have been induced through natural conditions and synthetic method. Naturally formed agarwood was obtained from broken and damaged tree trunk caused by natural phenomenon such as lightning, strong wind and insects gnawing on the tree trunk, and had eventually become rotten. Synthetically induced agarwood was produced by boring holes in the tree trunk using nails and filling the holes with an inoculation mixture that contained honey. The agarwood resin which was formed after five years was used in this study as the synthetically induced agarwood sample.
Experimental features	Essential oils were extracted from the trunk tissues of healthy and agarwood-containing trees using hydrodistillation (HS) method
Data source location	Hulu Langat, Selangor, Malaysia (3° 7′ 0″ North, 101° 51′ 0″ East)
Data accessibility	With the article
Related research article	A.A. Abd Rasib, F.X. Tong, Z.A. Mohamed-Hussein, R. Othman, Sequence analysis of terpene synthase cDNA from transcriptome profile of infected *Aquilaria malaccensis*, Malaysian Journal of Biochemistry and Molecular Biology 21 (1) (2018) 71-72

**Table:** 

**Value of the Data** • The metabolite datasets improve metabolomic database of agarwood-producing species including *A. malaccensis.* • The metabolomic data provide more information in understanding the formation of agarwood. • The data can be useful for comparative analysis with other synthetic inoculation methods used to induce agarwood production.

## 1 Data

The data presented here is a table showing the metabolite profiling analysis of agarwood essential oil obtained using GC-MS. The metabolite profiles were obtained from healthy tree trunk as control, and naturally formed and synthetically induced agarwoods. The dataset was tabulated in MS Excel (xlsx format) comprising compound name, retention time (RT), retention index (RI), molecular formula, molecular weight, fragments ion (*m*/*z*), significance value and normalised peak area. The data enhanced existing metabolite datasets for naturally formed and synthetically induced *A. malaccensis* agarwood which were obtained using two-dimensional GC coupled to accurate mass time-of-flight mass spectrometer (GCXGC-TOFMS) [1]. These data can be analysed together with transcriptomics data [2] to understand the metabolic or biosynthetic pathways in agarwood formation.

## 2 Experimental design, materials, and methods

### 2.1 Plant materials

The trunk from healthy A. malaccensis tree as well as those containing naturally formed and synthetically induced agarwoods were obtained from Hulu Langat (3° 7′ 0″ North, 101° 51′ 0″ East) in Selangor, Malaysia at 28 °C in the field. The plant samples were cleaned thoroughly, dried, cut into small slices, and ground mechanically.

### 2.2 Essential oil extraction

The essential oils for the non-resinous sample from the healthy tree and the resinous samples from the naturally formed and synthetically induced agarwoods were extracted using hydrodistillation (HS) method, following the method described with modification [3]. Ground agarwood (200 g) was soaked in distilled water (1 L) overnight and subsequently placed in a round-bottomed flask (2 L), with water volume adjusted to 1.5 L. For cooling purposes, the flask was connected to a clevenger type apparatus with tap water running. The sample was distilled for 5 h. The essential oil obtained was collected, separated using n-hexane and dried in vacuum rotary evaporator at 45 °C. The essential oil was placed in amber bottles and kept in −20 °C until further analysis. An internal standard, nanodecanoic acid, was added into the sample prior to GC-MS analysis.

### 2.3 Gas chromatography-mass spectrometry (GC-MS) analysis

All agarwood essential oils (in nine replicates each) were analysed on a PerkinElmer Clarus 600 GC-MS with total run time of 38.5 min (carrier gas helium, 1 mL/min, injection: split 20:1, 3 μL, injector and detector temperature of 200 °C and 300 °C respectively; oven temperature program: 60 °C for 3 min, 10 °C/min to 150 °C, held for 5 min, 10 °C/min to 285 °C, held for 8 min). The chromatographic separation was conducted using a PerkinElmer Elite 5MS capillary column (30 m × 0.25 mm ID x 0.25 μm, film thickness). Full scan mass spectra with mass range set from *m*/*z* 30 to 300 were obtained. Analysis of a series of *n*-alkanes standard solution (C8–C40) using the same GC-MS parameter was done to calculate the retention index (RI). All samples were injected continuously as one batch in random order.

### 2.4 Data handling

All GC-MS raw data were converted into NetCDF format and further processed using automated software for peak deconvolution (AMDIS). Compound identification was performed by comparing the ratios of mass-to-charge with a standard mass of NIST Mass Spectral 2008 library. Putative metabolites were assigned to peaks having similarity index of more than 70%. Peak alignment and peak area were obtained using Metabolomics Ion-based Data Extraction Algorithm, version 1.2.0 (MET-IDEA) software [4]. A dataset comprising compound name, retention time (RT), fragments *m*/*z* and the associated peak areas was obtained. Data normalisation was carried out using MetaboAnalyst 4.0 [5]. Briefly, peak area was normalised to the internal standard, nanodecanoic acid, followed by log transformation and subjected to data scaling using auto scaling. One way analysis of variance (ANOVA) (SPSS version 22) was used to calculate statistical significance for each metabolite.
